# The Thermophysical and Physicochemical Properties of the Aqueous Dispersion of Graphene Oxide Dual-Beam Thermal Lens Spectrometry

**DOI:** 10.3390/nano13142126

**Published:** 2023-07-21

**Authors:** Vladislav R. Khabibullin, Daria-Maria V. Ratova, Dmitrii N. Stolbov, Ivan V. Mikheev, Mikhail A. Proskurnin

**Affiliations:** 1 Analytical Chemistry Division, Chemistry Department, M. V. Lomonosov Moscow State University, d. 1, Str. 3, Lenin Hills, GSP-1, Moscow 119234, Russia; vladhab1995@gmail.com (V.R.K.); darmarrat@gmail.com (D.-M.V.R.); 2 Physical Chemistry Division, Chemistry Department, M. V. Lomonosov Moscow State University, d. 1, Str. 3, Lenin Hills, GSP-1, Moscow 119234, Russia; stolbovdn@my.msu.ru

**Keywords:** thermal lens spectrometry, graphene oxide, thermal diffusivity, reliability, photochemical reduction

## Abstract

Modern heat-conducting materials require special attention to analyze their thermophysical properties. Compared to classical methods, thermal lens spectrometry (TLS) has advantages due to its high sensitivity to physical and chemical composition. To avoid a systematic error in the analysis of complex systems, it is necessary to realize the limits of the applicability of the method. This study considers the features of thermal-diffusivity measurements by TLS in the stationary state for dispersed systems with absorbances up to 0.05. The limits of applicability of the method in analyzing heterogeneous systems are shown, and a mathematical apparatus is proposed for indicating a systematic error in finding thermal diffusivity that does not exceed 1%. Graphene oxide (GO), which has attractive physicochemical properties, was used as the object of analysis. GO belongs to 2D objects, the study of which requires highly sensitive methods and special attention when discussing the results. The thermophysical properties of aqueous dispersions of graphene oxide in a wide range of concentrations (up to 2 g/L) and lateral sizes (up to 4 µm) were studied by TLS. It has been found that with increasing nanophase concentration, the thermal diffusivity of graphene oxide dispersions passes through a minimum, which can be used in solving thermal insulation problems. It has been established that prolonged laser irradiation of the dispersion leads to a change in thermal diffusivity, which indicates the photochemical reduction of graphene oxide.

## 1. Introduction

In recent decades, the importance of the problems of heat transfer efficiency and heat storage has increased. The explosive development of electronics and microelectronics, increasing the power of devices, as well as the proper functioning of engines, power units, etc. (where the operating temperature must be maintained by continuous heat removal), have led to the need for new approaches to cooling and heat exchange. The increased number of publications on this topic evidences this; the literature shows that new heat-conducting materials and approaches to analysing thermophysical properties are required [1,2,3,4,5,6]. Classical cooling and heat-conducting materials (glycerin, water, glycols, metals, etc.) are not always effective. Liquid heat-conducting substances (water, ethylene glycol, etc.) have a high heat capacity. They can store thermal energy for a long time, but they dissipate it poorly due to low thermal conductivity. Extended surfaces (fins and microchannels), vibration, liquid suction/injection, and special/magnetic fields to improve heat transfer have come to a standstill. On the other hand, solid particles of millimeter or micrometer sizes have excellent thermal conductivity, and adding them to a base fluid increases heat transfer. However, the use of particles of this size causes problems. Dispersions have poor stability, leading to channel clogging [7].

One of the solutions to this problem is using heat-conducting nanofluids (NFs) [8,9,10]. Choi et al. [11] in 1995 found that the addition of a small amount of nanoparticles (NPs) to a solvent leads to a qualitative change in the thermophysical properties of pure solvents (water, ethanol, and ethylene glycol) and introduced a new term, nanofluids. 

Two groups of fillers are used to obtain heat-conducting NFs: metal-based and carbon nanomaterials. The first group includes metals (e.g., Cu, Ag, Au, Ag, etc. [12,13,14,15]), their oxides (ZnO, Fe_3_O_4_, TiO_2_, etc. [16,17,18,19,20]), quantum dots [21], nanocrystals [22], etc. The second group includes all carbon materials (nanotubes [23,24], graphene [25], carbon nanoparticles and dots [26], soot, etc.) [27,28]. The use of metal nanoparticles is complicated by dispersion stabilization as well as the chemical degradation of particles. Considering the surface modification of nanoparticles, obtaining stable dispersions in a wide range of concentrations is difficult [29]. The aggregation and sedimentation of nanoparticles in a dispersion worsen the thermal parameters of a nanofluid [19]. On the other hand, carbon nanomaterials (CNM) have more attractive properties, making them promising candidates for stable dispersions with desirable heat-conducting parameters [30]. Due to its developed surface and many functional groups, CNM has higher aggregative and sedimentary stability [31]. Due to the π–π-conjugation, carbon nanomaterials are more chemically inert than metal nanoparticles, which undergo chemical degradation in water [32,33]. Particular attention should be paid to the thermophysical properties, where carbon materials occupy a special place. The thermal conductivity of various CNMs at room temperature covers an extensive range of more than five orders of magnitude. For amorphous carbon, the thermal conductivity is ca. 0.01 W/(m·K). For diamond and graphene, it is more than 2000 W/(m·K); for carbon nanotubes, it is ca. 3000–3500 W/(m·K) [32,34]. For a type II diamond, the thermal conductivity can reach 10,000 W/(m·K) [34]. Compared to CNTs, metal nanoparticles have a lower thermal conductivity, reaching several hundred W/(m·K) [35]. The thermal conductivity of polymers is even lower, in the range of 0.1–0.5 W/(m·K) [36]. 

Graphene oxide occupies a special place among carbon nanomaterials. This is indicated by many studies, review articles, and monographs published over the past 5–7 years. To date, graphene oxide (GO) has already proven itself in solving medical problems (biosensors [37,38,39], bioelectronics [40], targeted drug delivery [41,42]) and environmental glitches (wastewater treatment [43,44,45]). As a classic 2D material, GO exhibits special electrically conductive properties and is successfully used to solve energy-related (supercapacitors [46]) problems [47]. Adding small amounts of GO to building materials qualitatively improves their physical and mechanical properties. With the addition of graphene oxide, new types of cement [48,49], anticorrosion and refractory materials, coatings [50,51], and polymer composite materials with variable properties [52] are obtained. In addition, GO has a high thermal conductivity [53,54], which makes it a promising candidate as a filler for heat-conducting nanofluids. At the same time, despite its many valuable properties and wide range of applications, there are currently no systematic studies of the thermophysical properties of aqueous dispersions of graphene oxide in a wide range of concentrations and sizes. 

Various fillers for heat-conducting NFs and their morphological and physicochemical features require new approaches to analyzing thermal parameters. In reviews devoted to heat-conducting nanofluids, the authors point out contradictions in measuring the same or similar systems regarding physicochemical and morphological properties [55,56]. This issue requires the unification and standardization of approaches to analyzing complex heterogeneous systems. At the same time, for heat-conducting NFs, it is necessary to measure not only thermal conductivity (*k*), heat capacity (*C_p_*), viscosity (*η*), and density (*ρ*) but also thermal diffusivity (*D*). Classical methods for measuring thermal parameters show low sensitivity to the nanophase, which makes these methods unsuitable for thermal parameters and thermal diffusivity in particular [57].

One of the solutions to this problem is the use of photothermal spectroscopy (PTS). The group of photothermal methods is based on registering the nonradiative relaxation of excited molecules. It has proven to be a fast and highly sensitive approach to analyzing the thermophysical properties of liquids, solids, and complex systems [58,59,60]. Among the methods of PTS, a special place is occupied by thermal-lens spectrometry (TLS), which is used to analyze the thermophysical properties of various dispersed systems and heat-conducting nanofluids [19,61,62,63]. TLS is highly sensitive to low concentrations of the solid phase and differences in the morphological properties of the nanophase [21,26,27,64]. Using TLS, differences in the thermophysical properties of bimetallic nanoparticles with different ratios of metals were determined [65]. In another study [66], TLS displayed significant changes in the temperature diffusivity of particles in a narrow size range of 4.6–5.2 nm. It is worth noting that the measurement of thermophysical properties is one of the applications of TLS. Due to the possibility of continuous measurement and recording of transient curves, the method is successfully used to analyze the kinetics of physical and chemical processes, in the analysis of photochemical reactions, in flow analysis, as a detector for high-performance liquid chromatography, etc. [67] 

However, despite the significant advantages of TLS in analyzing heterogeneous systems, measurement accuracy issues require special attention. Additionally, in many cases, the results of TLS cannot be applied to other systems. This significantly limits the applicability of TLS. Earlier papers devoted to the influence of systematic errors on the determination of thermophysical parameters for homogeneous systems [68] pose questions regarding the correctness of measurements for heterogeneous systems [57]. In [57], general recommendations for thermal-lens measurements were proposed for low-absorbing silicon oxide nanoparticles. 

In this study, the thermophysical properties of aqueous dispersions of graphene oxide were measured by dual-beam thermal lens spectrometry in a wide range of concentrations and sizes of the nanophase. Primary attention is paid to validating thermal diffusivity measurements as a key issue in analyzing dispersions with high absorbances. In conclusion, the influence of continuous long-term laser radiation on the physicochemical and thermal properties of the GO–water system is considered. The results of this study make a contribution to the development of thermal lens spectrometry. This article will be helpful for researchers involved in the analysis of the thermophysical and physicochemical properties of complex systems. The research results expand the understanding of the thermophysical properties of aqueous graphene oxide dispersions and can help solve problems in heat engineering, energy, and materials science.

## 2. Materials and Methods

### 2.1. Preparation of Graphene Oxide Dispersions

The aqueous graphene oxide (Hummers’ type) (LLC Rusgraphene, Moscow, Russia) dispersions were prepared with extra exfoliation, including ultrasound probe usage (25 mL, 2 g/L). Purification procedures have been conducted elsewhere, published in [69]. The sized fractions of graphene oxide were prepared using the standard protocol published elsewhere [70] with slight modification using another membrane type. The stock solution of graphene oxide was purified using a 0.5 kDa membrane (dialysis bag). The following fraction was prepared: bulk >0.5 kDa, >14 kDa, 0.5–14 kDa, 3.5–14 kDa, 3.5–1.0 kDa, and 0.5–1.0 kDa after preliminary purification from: (1) oxidizing agents for synthesis GO such as KMnO_4_, H_2_SO_4,_ etc., and (2) oxidation debris (OD) [71]. The SEM images of the graphene oxide fraction are presented in Figure S2 (Supplementary Materials).

### 2.2. Thermal-Lens SPECTROMETER

A dual-beam thermal lens spectrometer was used. Optimization of the spectrometer and measurement parameters is presented in [57,68]. The scheme and description of the spectrometer are explained in detail in the Supplementary Materials (Figure S1 and Procedures section, thermal-lens spectrometer). Measurement parameters are presented in Table S1.

### 2.3. Thermal-Lens Measurements

The Shen–Snook model presented in the Supplementary Materials (Procedures, Shen–Snook model) was used to describe thermal lens measurements. This section shows the adaptation of the model to measurements of heterogeneous systems and represents the equation needed to find the thermal diffusivity of heterogeneous systems. Below are the basic equations necessary to find the thermal diffusivity of GO dispersions with slight changes. 

For ease of use of Equation (S1), we introduce substitutions and represent them as:

(1) $$I\left(t\right)=I\left(0\right){\left[1-0.5\theta {tan}^{-1}\left(a/\left(b\left(t_{c}/2t\right)+c\right)\right)\right]}^{2},$$

where *a*, *b*, and *c* are constants, *a* = 2 *mV*, *b* = (1 + 2 *m*)^2^ + *V*^2^, *c* = 1 + 2 *m* + *V*^2^, respectively. As proposed in [57], Equation (1) is presented as a function of the effective characteristic time $\tilde{t_{c}}\left(t\right)$ at each moment of the transient development curve:

(2) $$\tilde{t_{c}}\left(t\right)=[(a/tan[2\times (1-\sqrt{I\left(t\right)/I\left(\infty \right)})/\theta^{\prime }])-c]\times 2t/b.$$

Here, $\theta^{\prime }$= 2[1 − *I*(∞)/*I*(0)]/tan^−1^(*a*/*c*), where $I\left(\infty \right)$ is the stationary-state probe intensity. This study uses the full development of the thermal field and the achievement of a stationary state. The data for the last 300 ms of the transient curve were averaged for homogeneous and heterogeneous systems with no thermophoresis. For the transition from effective to the true value of $t_{c}$, the first 150 ms of the function $\tilde{t_{c}}\left(t\right)$ were averaged, after which, using Equation (S5), the thermal diffusivity of the system was found. 

When pronounced thermophoresis is present in disperse systems, the characteristic time and thermal diffusivity are determined according to the recommendations in [57]. In this case, the corrected steady-state probe laser intensity $I^{\prime }(\infty )$ is used, which is found by fitting the first 150 ms of the theoretical transient curve with the experimental one so that the first 150 ms correlate well. 

In this case, the characteristic time can be found according to Equation (1) using the corrected intensity $I^{\prime }(\infty )$:

(3) $$\tilde{t_{c}}{}^{\prime }\left(t\right)=[(a/tan[2\times (1-\sqrt{I\left(t\right)/I^{\prime }\left(\infty \right)})/\theta^{\prime }])-c]\times 2t/b,$$

The transition from effective to true characteristic time was also carried out by averaging the first 150 ms of the function $\tilde{t_{c}}{}^{\prime }\left(t\right)$, after which, using Equation (S5), the thermal diffusivity of the system was found.

Transient thermal-lens curves in this study are used in two normalized forms. In the first form, normalization is made using the largest value *I*(*t*)/*I*(0). In the second, the normalization was performed as

(4) $$\tilde{I}\left(t\right)=\frac{\left[I(t)–I(\infty )\right]}{\left[I(0)–I(\infty )\right]},$$

where I(∞) is the intensity of the probe beam in the stationary state. Depending on the presence/absence of thermophoresis, I(∞) is found either by fitting (in which case it is $I^{\prime }(\infty )$), or by averaging the last 300 ms of the transition curve (true $I\left(\infty \right)$). This normalisation form makes distinguishing between the thermal lens and thermophoresis processes possible.

In addition to thermal diffusivity, a thermal lens signal was used as an analytical signal, which is found according to the following equation:

(5) $$\vartheta =\frac{I\left(0\right)-I\left(\infty \right)}{I\left(0\right)}$$

Here, the transient curve average of the last 300 ms was used as the steady state. Since the excitation laser range of absorbances and powers were widely varied, the thermal-lens signal in two forms was used for comparison. In the first form, the thermal lens signal was normalized to the excitation radiation power (ϑ/P). In the second form, the signal was normalized concerning power and absorbance, obtaining ϑ/(PA).

To compare the normalized thermal-lens signals with each other, an additional normalization 1 ÷ 0 was carried out while obtaining $\tilde{\vartheta }/P\;$ and $\tilde{\vartheta }/PA$, where the normalized thermal lens signals ϑ/P  and ϑ/(PA) at the highest concentration of GO were taken as unity. 

Random errors in finding the thermal diffusivity and thermal lens signal were carried out according to known equations presented in detail in the Supplementary Materials (see Procedures section, Statistical analysis).

### 2.4. Other Instruments and Their Operation Parameters

ATR-FTIR spectra for GO samples were recorded on a Bruker Vertex 70 single-beam IR Fourier spectrometer (Bruker Optik GmbH, Ettlingen, Germany) equipped with a GladiATR™ (PIKE Technologies, Fitchburg, WI, USA) monolithic diamond ATR for the entire spectral range from 4000 to 400 cm^−1^.

The morphology and structure of the samples were studied using a JEOL JSM-6390 LA scanning electron microscope (SEM) (JEOL, Tokyo, Japan). Samples were freeze-dried, placed on a double-sided carbon conductive tape, pumped, and checked at an acceleration voltage of 20 kV. The samples were preliminarily frozen for freeze-drying at a temperature of −60 °C. Frozen samples were dried in vacuo at temperatures of 3, 10, and 20 °C for 24 h.

UV/visible absorption spectra were recorded using a Cary 4000 scanning double-beam spectrophotometer (Varian, Mulgrave, Australia). Spectrophotometric measurements were carried out in Agilent Quartz Cuvettes with an optical path length of 1 mm and a registration pitch of 1 nm in the 190–800 nm range.

## 3. Results

### 3.1. Verification of Correct Operation of the Spectrometer

**Figure 1:**
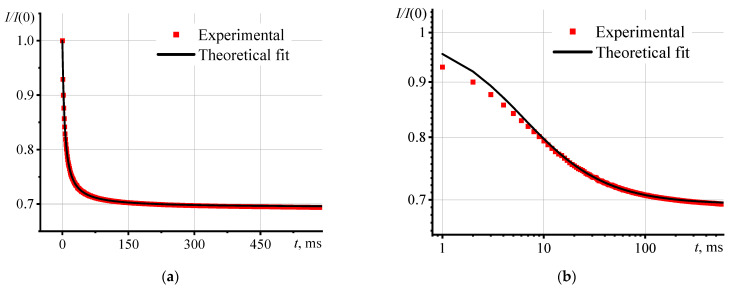
Transient curves for an aqueous solution of Ferroin (1 μmol/L) in linear (**a**) and logarithmic (**b**) scales.

To confirm the correct operation of the spectrometer, the model and measurement conditions, before analyzing aqueous graphene oxide dispersions, the thermal diffusivity of an object with precisely known thermal and optical properties was measured. A pure solvent (ethanol, toluene, acetonitrile, water, etc.) is usually used as a reference material [28]. The research objects are aqueous graphene oxide dispersions, and pure water was used as a reference. Figure 1 shows the transient curves (normalized form, according to Equation (4)) of an aqueous solution of a photostable dye, Ferroin, with a concentration of 1 μmol/L.

The transient curves of the model approximation clearly describe the experimental transition curves. The average (*n* = 5) thermal diffusivity (Equation (S9)) was 0.143 mm^2^/s, which agrees well with the reference data [72]. The low relative standard deviation (Equation (S7), 2.4%) indicates high reproducibility of the results and a low random error. Thus, the thermal lens spectrometer works correctly, and the selection of operating measurement parameters is optimal. Hence, all deviations that were observed for dispersions of graphene oxides are related to the features of the samples.

### 3.2. Effect of Graphene Oxide Concentration and Size on the Thermophysical Properties of Aqueous Dispersions

**Figure 2:**
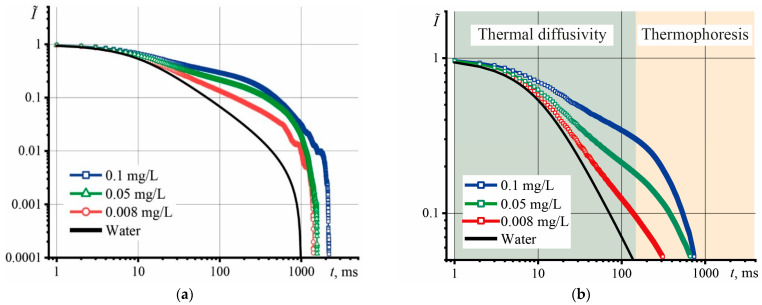
Transient curves of aqueous dispersions of GO, fraction > 14 kDa with different concentrations, where (**a**) complete transient curves, and (**b**) the initial segment of transient curves.

The size and concentration of the nanophase are the key parameters that affect the thermal properties of heat-conducting nanofluids [56]. The logarithmic coordinates of transient curves make it possible to identify thermal effects (in addition to thermal lens) that occur in disperse systems, as recommended in [57]. Figure 2a shows the transient heating curves, using the example of the largest fraction of GO for several concentrations. 

The shape of the transient curves demonstrates the presence of thermophoresis, which increases its influence with increasing concentration. An increase in the characteristic time occurs before the onset of thermophoresis, which appears after 150–200 ms and does not affect the determination of *D* (Figure 2b). The section up to 150 ms is responsible for thermal diffusivity and is used to determine *D* with a minimum systematic error. The behavior of the transient curves of GO dispersions (Figure 2a) indicates slow heating and a longer time to reach the steady state. As the concentration increases to 0.1 mg/L, the time to reach the steady state increases, which indicates a longer time to reach thermal equilibrium. Hence, it can be assumed that GO dispersions exhibit good thermal insulation properties at a concentration level of 0.1 mg/L.

**Figure 3:**
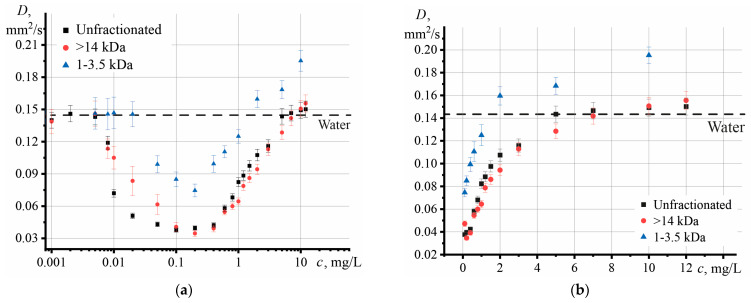
Effect of graphene oxide concentration on thermal diffusivity for an unfractionated sample and two fractions: > 14 kDa and 1–3.5 kDa (*n* = 5, *p* = 0.95), where (**a**) over the entire concentration range, (**b**) at *c* > 0.1 mg/L.

The found thermal diffusivity (using Equations (2), (3) and (S5)) showed (Figure 3a) that all GO fractions demonstrated similar behavior with increasing concentration. As the concentration of the nanophase in the dispersion increased to 0.1 mg/L, the thermal diffusivity decreased. The lowest value was observed at 0.1 mg/L. A further increase in the graphene oxide concentration led to an increase in thermal diffusivity. After 2–3 mg/L, all samples demonstrated a decrease in the rate of increase in *D* and reached a constant value (Figure 3b).

**Figure 4:**
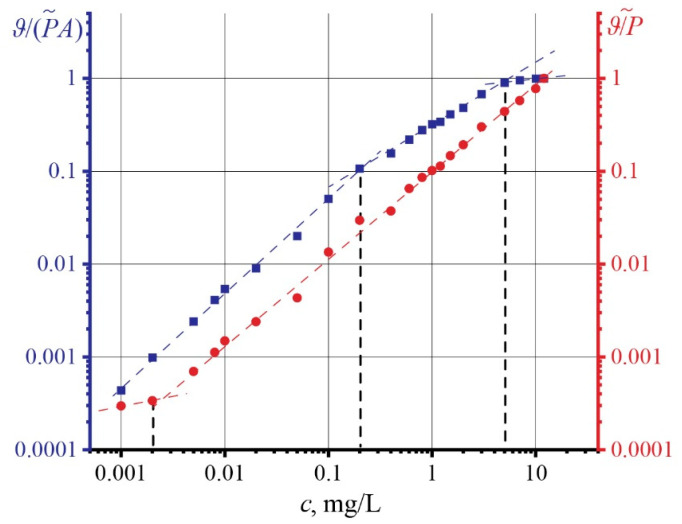
Dependence of normalized thermal lens signals on concentration for unfractionated GO.

Figure 4 shows the dependences of the concentration of unfractionated GO on the thermal lens signal (found by Equation (5)) in two forms: red is the ratio of the thermal lens signal to the power of the excitation laser *ϑ*/*P*, and blue is the ratio of the thermal lens signal to the power and absorbance of the samples *ϑ*/(*PA*). It was found that the *ϑ/P* signal curve changes the nature of its dependence with increasing concentration at *c* > 0.005 mg/L, where a linear increase for *ϑ*/(*PA*) is observed. On the other hand, three regions can be distinguished for the thermal lens signal ϑ/(*PA*) with increasing concentration. The first change in linearity is observed at a concentration of 0.2 mg/L. At *c* > 7 mg/L, the second change in the dynamics of the signal occurs, where the signal reaches a constant value, repeating the dynamics of thermal diffusivity. 

**Figure 5:**
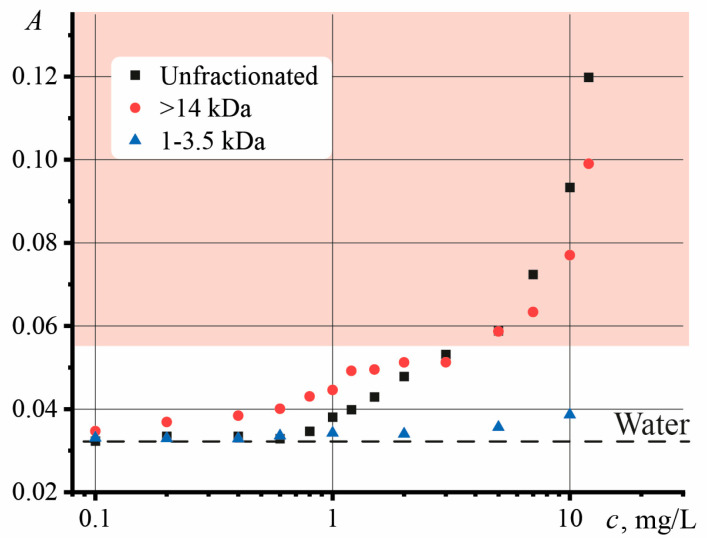
Dependence of the absorbance of GO samples with increasing concentration. The red zone corresponds to the significant influence of the systematic error on finding the thermal diffusivity.

Figure 5 shows the dependences of the absorbance of the GO samples on the concentration, where the red rectangle corresponds to the area of the significant influence of the systematic error and is outside the performance limits of the Shen–Snook model. 

**Figure 6:**
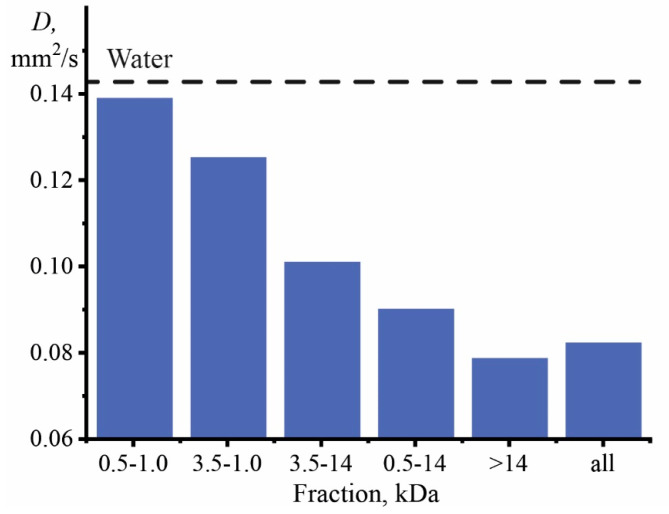
Effect of graphene oxide size on the thermal diffusivity of an aqueous dispersion at a solid phase concentration of 1 mg/L (*n* = 3, *p* = 0.95).

The influence of graphene oxide size on thermal diffusivity showed that, compared to pure water, samples of different fractions demonstrate a decrease in thermal diffusivity (Figure 6).

The largest particles make the most considerable contribution to the decrease in thermal diffusivity, and the smallest value of *D* is observed for the GO fraction of >14 kDa. The 0.5–1 kDa fraction showed the highest thermal diffusivity of 0.139 mm^2^/s, which is close to the value of pure water. The relative standard deviation *s_r_* for all samples did not exceed 5%, and in the case of the initial GO, it was 2.1%.

### 3.3. Influence of an Excitation Laser on the Physicochemical and Thermal Properties of GO

Continuous measurement of the thermal lens signal and thermal diffusivity makes it possible to use TLS to analyze long-term processes and to register the dynamics of physicochemical and photochemical reactions occurring in the system [73,74]. For disperse systems, a change in thermal diffusivity may indicate aggregation/agglomeration or, on the contrary, decay and destruction. In this regard, the long-term behavior of GO under the action of radiation is of interest. We used thermal diffusivity and the thermal lens signal as analytical signals. The analysis was carried out for more than four days. The largest fraction of graphene oxide (>14 kDa) with a concentration of 0.1 mg/L was used. In this case, averaging was carried out over 600 transition curves (1 h of measurements).

**Figure 7:**
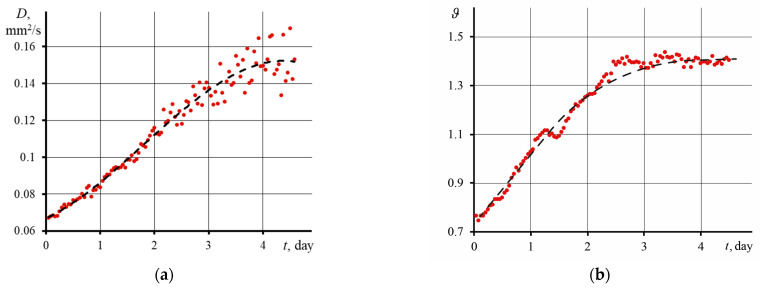
Changes of (**a**) thermal diffusivity and (**b**) the thermal lens signal with time for GO (fraction > 14 kDa) at a concentration of 1 mg/L.

Figure 7 shows graphs of thermal diffusivity (a) and thermal-lens signal (b) versus time. The thermal diffusivity of dispersions (found by Equation (S5)) increases with irradiation time. During the first three days, a linear increase in thermal diffusivity was observed, after which the trend changed, and thermal diffusivity reached a constant value.

Similar dynamics were observed for the thermal lens signal (found using Equation (5)), where after three days of measurements, *ϑ* reached a constant. For thermal diffusivity, after three days, an increase in the spread of *D* relative to the average was observed (dashed line), which was not observed for the thermal lens signal. This indicates that the observed fluctuations result from physicochemical processes in the system that affect the development and shape of transient curves. 

**Figure 8:**
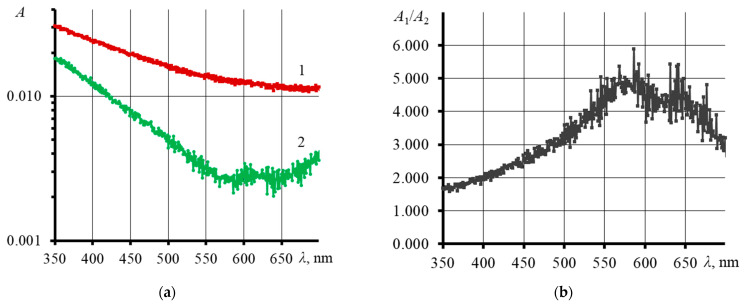
(**a**) Absorption spectra of graphene oxide (>14 kDa, *c* = 1 mg/L), before (1) and after (2) a long-term thermal lens experiment; (**b**) ratio of absorbance for GO after laser irradiation to absorbance before irradiation.

After a long-term thermal-lens experiment, an increase in absorbance was observed (Figure 8a,b) and a change in the GO UV spectra. This indicates changes in the chemical structure of GO and an increase in the number of particles in the system.

Below are the IR spectra of graphene oxide samples before and after prolonged irradiation.

**Figure 9:**
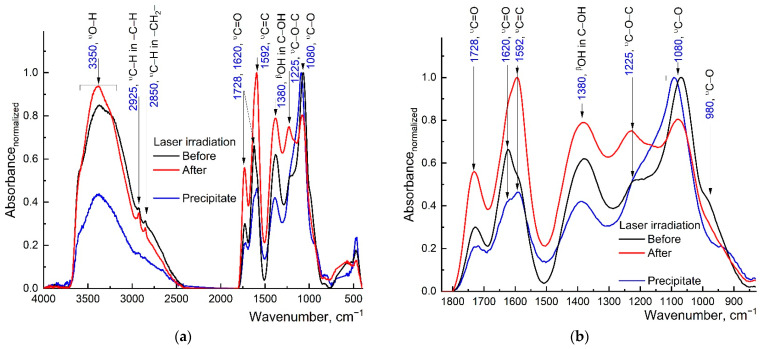
ATR FTIR spectra for the graphene oxide samples before (solid black line) and after (solid red line) the laser irradiation. The solid blue line is for the sample, which had precipitated during laser irradiation for four days: (**a**) in the range of 4000 to 400 cm^−1^, and (**b**) the fingerprint region.

The overall ATR-FTIR spectrum of graphene oxide samples before and after laser treatment and sedimented graphene oxide samples is given in Figure 9a. The broad absorption band in the region of 3600–2400 cm^−1^ includes the absorption band of water [75], ν_O–H_ fragments (3350 cm^−1^), and ν_C–H_ (–CH at 2925 cm^−1^, –CH_2_– at 2850 cm^−1^). Overlapping bands of oxygen groups in the fingerprint region (Figure 9b), which refer to ν_C=O_ (1728 and 1620 cm^−1^) in ketone and acidic forms, aromatic ν_C=C_ (1592 cm^−1^), β_O–H_ (C–OH 1380 cm^−1^), ν_C–O–C_ (1225 cm^−1^), and ν_C–O_ (1080 cm^−1^), are also observed [76]. In the fingerprint region, one can observe that the laser treatment of aqueous dispersions of graphene oxide increased the intensity of the ν_C=C_ band (1592 cm^−1^) and decreased the intensity of the ν_C=O_ (1728 and 1620 cm^−1^), and ν_C–O_ (1080 and 980 cm^−1^) bands, which may indicate the formation of reduced –OH moieties [77]. It should be noted that the intensity of the ν_C–O–C_ band (1225 cm^−1^) did not change relative to the others, which indicates the preservation of the graphene oxide plane (likely fewer defects).

## 4. Discussion

### 4.1. Effect of Graphene Oxide Concentration and Size on the Thermophysical Properties of Aqueous Dispersions

To explain the results of the concentration and size dependences of thermal diffusivity obtained in this work, it is necessary to consider the effects and physical principles that affect heat transfer in dispersed systems.

According to the well-known relation *D* = *k*/*ρC_p_*, thermal diffusivity is proportional to thermal conductivity and inversely proportional to density and heat capacity. An increase in the content of a nanophase in a nanofluid, as a rule, leads to an increase in density and thermal conductivity as well as a decrease in the heat capacity of the system [78]. Zhou et al. [79] demonstrated that for nanofluids in low concentrations, in a narrow range of concentrations, the volumetric heat capacity (product *ρC_p_*) is a constant. In this work, GO dispersion densities and specific heats were not measured, so it cannot be approved that *ρC_p_* is a constant. However, the literature analysis allows us to draw the following conclusions. To obtain nanofluids, as a rule, concentrations above ca. 0.1 vol.% are used, where noticeable changes in heat capacity, density, and viscosity are observed [78,80,81]. In such a case, dispersions with nanophase concentrations of <1 × 10^−4^ vol.% are used. This makes it possible to neglect a change in heat capacity and density in GO dispersions and assume that *ρC_p_* = const.

Following further considerations, the thermal diffusivity of a dispersion largely depends on thermal conductivity and, as shown below, often repeats its behavior. The mechanism of thermal conductivity of nanofluids and an aqueous dispersion of graphene oxide is complex and multifactor [56]. To explain the results obtained, it is necessary to briefly consider the main mechanisms of thermal conductivity in GO dispersions, which are currently distinguished in the literature. Being a solid material, graphene oxide shows the main mechanism of the thermal conductivity of lattice vibrations (phonons) and the movement of free electrons [82,83]. The scattering of primary energy carriers at the boundary is called phonon scattering. As the size of the material decreases, the surface area and the interfacial boundary increase significantly. This leads to an increase in phonon scattering and heat transfer efficiency. However, as the size of the material decreases below 35 nm (phonon mean free path), the intrinsic thermal diffusivity of NPs decreases, and the efficiency of heat transfer decreases [81].

For liquids (particularly water), thermal conductivity is mainly due to Brownian motion (molecular diffusion). Hence, the thermal diffusivity of the GO nanofluid depends on the phonon-transport properties, the motion of free electrons, and the Brownian motion [27]. For nanofluids, several additional factors are distinguished, among which are: thermophoresis, thermal conductivity at the nanophase-liquid interface, where the surface area of the nanophase affects, and alignment of the transverse temperature gradient in the liquid due to nanoparticles [29,84].

An analysis of the existing data has shown that, in many cases, the presence of carbon nanomaterials in large quantities (mg/mL level) increases the thermal conductivity of the dispersion [28,84,85,86]. As previously shown [27,78,87,88,89,90], an increase in the GO concentration leads to an increase in *k* of the dispersion, which is the main reason for the increase in thermal diffusivity.

Rarely increasing the nanophase concentration leads to a decrease in thermal diffusivity. Here, it is difficult to identify the main cause common to all. In each case, it has its own. Thus, in the case of the toluene–InP system, the decrease in thermal diffusivity is due to low *D* of InP nanocrystals themselves [22]. The authors pointed out that clustering and aggregation, on the contrary, contributed to an increase in phonon transport properties and an increase in *D* [22]. In [26,91], the reason for a decrease in thermal diffusivity, as noted by the authors, is the enhancement of the phonon-phonon interaction in the medium. In another study [92], the decrease in thermal diffusivity at low concentrations was explained by the influence of osmophoretic motion, and the increase in thermal diffusivity, which was observed in the range of high concentrations, was explained by Brownian motion, which began to play a major role. Attention is also drawn to the fact that high concentrations lead to radiation scattering, which distorts the value of *D* [92]. 

The dependences presented in Figure 3 reflect the effect of the mass concentration of GO on thermal diffusivity. On the other hand, the fractions have different molecular weights of GO, and it is necessary to use a correction for the number of particles in each sample.

Using these recommendations, several conclusions can be drawn:

(1) The largest particles contribute the most to the decrease in thermal diffusivity;
(2) The contribution of particles with different molar masses to the increase in thermal diffusivity, which was observed at *c* > 0.1 mg/L, is comparable with each other.

At the same time, the first position is also confirmed by the results presented in Figure 6. An increase in particle size leads to a decrease in Brownian motion, which worsens thermal diffusivity [21]. Significant Brownian motion is observed for smaller particles, leading to slight thermal diffusion [56]. This was observed previously for quantum dots [21] and some nanoparticles [66]. In [93], for nickel-oxide nanoparticles coated with PVP, a slight increase in size (from 6.9 to 8.9 nm) led to a significant decrease in *D* (by three times). The same dynamics are demonstrated by the thermal conductivity of dispersion, which, as a rule, decreases with an increase in the nanophase size [78]. For graphene, it was found [25] that single-layer graphene has the highest thermal conductivity, and with an increase in the number of atomic layers, thermal conductivity approaches the value for bulk graphite.

However, it is necessary to make a small digression and note that an increase in the size of a nanophase in a dispersion, as a rule, leads to an increase in the thermal conductivity of a nanofluid. This behavior is typical for metal nanoparticles [15,18,19,57,94,95,96], where the largest particles make the greatest contribution to the thermal diffusivity of the system. This can be caused by clustering effects at high concentrations, which contribute to an increase in thermal conductivity [56].

The change in the dynamics of thermal diffusivity, which was observed at *c* > 0.1 mg/L (Figure 3b), may indicate a difference in the heat transfer mechanism. With increasing concentration, the thermal conductivity of the particles begins to play a significant role, which significantly enhances its effect due to the possible agglomeration of GO [56]. At the same time, we do not consider the extreme case, when a high concentration leads to precipitation and a decrease in *k*. The behavior of the thermal-lens signal *ϑ*/(*PA*) also exhibits a change in dynamics at a ca. 0.2 mg/L concentration. Signal normalization to both power and absorbance makes obtaining the contribution of only thermophysical properties possible. The thermal lens signal *ϑ*/(*PA*) repeats the behavior of the dependence of thermal diffusivity on concentration. This makes *ϑ*/(*PA*) an additional analytical indicator of thermal processes in the system.

Of interest are the results of measurements of high concentrations of GO. Here, a change in the increase in thermal diffusivity rate was found for *c* > 5 mg/L. Thus, two questions arise: Is the found value of thermal diffusivity true, and the change in the linear growth of *D* vs. *c* dependence at high concentrations a consequence of the thermal effects of the system (for example, clustering and a decrease in Brownian motion)? Or is the found thermal diffusivity incorrect, and do the observed changes in growth rate *D* increase the systematic error? Thus, the question of the correctness of determining *D* for strongly absorbing systems is a key one and will be considered in detail below.

### 4.2. Influence of High Optical Absorption on the Reliability of Finding the Thermal Diffusivity 

Despite the high sensitivity of TLS to detecting changes in the composition of the medium and the precision of the obtained values of thermal diffusivity, the question of the reliability of *D* measurements requires special attention. Previously, questions of the quantitative influence of systematic errors on determining the thermal diffusivity of pure solvents were considered [68]. The questions of the correctness of *D* measurements for dispersed systems were also assessed using an example of weakly absorbing SiO_2_ nanoparticles [57]. Here, the questions of the correctness of measuring the thermal diffusivity for dispersions with high optical absorption are considered using an example of dispersions with the largest fraction and unfractionated graphene oxide. 

The change in the dynamics of the concentration dependence of thermal diffusivity at *c* >5 mg/L can have two causes: thermophysical and instrumental. The first reason may be the deterioration of the Brownian motion of graphene oxide particles at high GO concentrations. Previously [16], it was found that with increasing concentration, the thermal diffusivity reaches a maximum and then decreases, which the authors attributed to the deterioration of Brownian motion due to a high concentration of the nanophase. Additionally, an increase in the GO concentration leads to particle agglomeration, which also reduces the efficiency of thermal diffusivity due to a decrease in the Brownian motion [56].

The second reason may be related to the limitation caused by the high absorbance of the sample [97]. As noted in [98], an increase in the absorbance leads to an increase in the apparent thermal diffusivity. Similar dynamics have been observed previously [68]. It was shown that in TLS, it is necessary to use colored solutions with an absorbance no higher than 0.05. Absorbances above this value contribute to an increase in systematic error, manifested as an increase in the found thermal diffusivity. In our case, the absorbance for the unfractionated and largest GO concentration above 3 mg/L (Figure 5, red zone) is higher than the threshold value, which may adversely affect the reliability of finding *D*. 

To confirm the second reason, let us consider the dynamics of the thermal lens signal changes. Figure 4 shows the effect of the concentration of unfractionated GO on the thermal-lens signal in two forms. The sample selection is associated with a low random error (using Equation (S7)) in determining the thermal diffusivity. It can be seen from the results that after 0.005 mg/L, the normalized thermal-lens signal *ϑ*/*P* changes. At the same time, the signal *ϑ*/*PA* in the same region demonstrates a linear increase with increasing concentration. At high concentrations, the shape of the thermal lens signal *ϑ*/*P* is disadvantaged. *ϑ*/*P* retains its linear dependence up to the limiting power level of the spectrometer and the highest absorbance while going beyond the model applicability. 

Here, it is necessary to use the thermal lens signal *ϑ*/*AP*, which allows for considering absorbance. As can be seen from Figure 4, in the region of *c* >7 mg/L, there is a change in the dependence and reaching a constant, repeating the dynamics of thermal diffusivity. With a reliable measurement of the absorbance and power of the excitation beam, the observed changes in the thermal lens signal *ϑ*/*AP* are caused by the limitations of the TLS. 

Thus, it can be concluded that the thermal diffusivity for a GO sample with a concentration above 7 mg/L has a high systematic error caused by the optical limitations of the Shen–Snook thermal-lens model. As a recommendation in the analysis of highly absorbing disperse systems by TLS, it is proposed to use a normalized thermal-lens signal concerning power and absorbance as a criterion of correctness. This will make it possible to reveal the limits of applicability of the method in analyzing heterogeneous systems and reduce the systematic error. 

As for the dynamics of the *D* vs. *c* dependence for the smallest fraction of GO, in this case, absorbance over the entire range of concentrations is within the acceptable absorbance limits. In this case, as mentioned above, the assessment of the effect of GO on thermal diffusivity, corrected for the number of particles, is approximately the same. A decrease in the rate of change in thermal diffusivity here may be caused by the deterioration of the Brownian motion due to a high concentration of particles, which leads to a deterioration in heat transfer.

### 4.3. Influence of Laser Radiation on Physicochemical and Thermal Properties of Graphene Oxide Dispersion

After prolonged irradiation, a black precipitate was found at the bottom of the cell. Provided that the dispersion of graphene oxide is stable for several months, the precipitate formed indicates changes in the physicochemical nature of graphene oxide. The behavior of the thermal diffusivity, thermal lens signal, UV (Figure 8a,b), and IR spectra (Figure 9a,b) of GO may indicate the destructive effect of laser radiation and the decomposition of GO into separate layers. After two days of irradiation, as seen in Figure 7a, the spread in thermal diffusivity increases from the average (Figure 7a, dotted line). In such long-term experiments, fluctuations and convection effects appear, which have a longer period. The relative standard deviation (Equation (S7)) for the initial thermal diffusivity values was less than 0.3%, and by the end of the analysis, RSD increased by an order of magnitude and amounted to more than 3%. This low random error eliminates random fluctuations and noise affecting the measurement result. In other words, the observed increasing amplitude of thermal diffusivity is a consequence of ongoing changes in the GO dispersion under the action of laser irradiation. A possible explanation for this could be the following. Over time, in the irradiation zone of the excitation beam, products of decomposition and degradation of GO accumulate, which contribute to the value of *D*. At the same time, it is still difficult to guess which chemical process prevails. Thermophoresis begins to appear, periodically renewing the irradiation zone, and transferring the products of GO transformations outside this zone. Hence, a large spread in thermal diffusivity is observed: the largest *D* refers to the effects of chemical degradation and decomposition of GO, and the smallest *D* to graphene oxide, where the changes occurred to a lesser extent.

In confirmation of these assumptions, several works in the literature are committed to GO chemical transformations under laser radiation and oxidants. As noted in [99], for 2D materials, the mutual arrangement of layers is of great importance. Material properties can vary greatly depending on the layered packing. At the same time, in the coating itself, there may be no changes at all (in chemical bonds, functional groups, etc.). Many 2D materials show eliminating oxygen-containing groups under laser or thermal action [99].

The method of GO recovery is based on the use of lasers (usually with UV radiation) [100]. As noted in [101,102,103], UV radiation decreases the size and changes the structure of graphene oxide by removing functional oxygen groups. The decrease in the intensity of the bands of oxygen-containing groups in our case (Figure 9b) confirms the occurrence of a photochemical reduction reaction. It was also shown in [104] that UV radiation (*λ* = 266 nm) effectively reduces the oxygen content in GO, reducing it, while IR radiation (*λ* = 1064 nm) more effectively promotes transformation into a typical *sp*^2^-carbon structure of graphene. Of interest is the study in [105], where it was found that GO, under the action of irradiation with a wavelength of 440 nm and in the presence of FeCl_3_ and H_2_O_2_, underwent significant oxidation and decomposition: the number of layers decreased by more than an order of magnitude.

In the review in [34], it was said that with an increase in the number of layers, the thermal conductivity decreases, approaching the graphite value, and even drops lower at *n* ≈ 8. The degree of oxidation can also significantly affect the thermal characteristics of graphene oxide [106]. A low degree of oxidation improves the phonon transport properties of GO [106]. Oxygen functional groups, on the contrary, reduce the efficiency of phonon transfer in GO and adversely affect the thermal characteristics. The calculated thermal conductivity of graphene oxide at room temperature is about 72 W/(m·K) at an oxidation state of 0.35 and ca. 670 W/(m·K) at an oxidation state of 0.05 [106].

Thus, taking into account the previous fact that a decrease in oxygen groups contributes to an increase in thermal conductivity [106,107] as well as the literature data described above, we can conclude that two processes occur in this system under the action of radiation: GO decay and its (photo)reduction.

## 5. Conclusions

In this study, thermal lens spectrometry was used to consider the thermophysical properties of the aqueous dispersion of graphene oxide in a wide range of concentrations and sizes. It was found that the largest fraction of graphene oxide has the greatest effect on thermal diffusivity. All considered fractions demonstrate a significant decrease in thermal diffusivity with an increase in the amount of nanophase, reaching a minimum of thermal diffusivity at concentrations of ca. 0.1 mg/L. A further increase in the graphene oxide concentration increases the dispersion thermal diffusivity. Thus, graphene oxide is a promising candidate as a filler in heat-conducting nanofluids for heat-insulating purposes. Prolonged exposure of the dispersion to laser radiation leads to the decay and reduction of graphene oxide, increasing the thermal diffusivity of the dispersion. In this paper, questions regarding the correctness of measuring the thermal diffusivity of heterogeneous systems with high optical absorption were considered. The proposed approaches, analysis methodology, and processing of thermal lens spectrometry results make it possible to reveal the limitations regarding the applicability of the method and avoid a high systematic error in the analysis of the thermal diffusivity of heterogeneous systems with high optical absorption.

## Data Availability

Not applicable.

## Supplementary Materials

The following supporting information can be downloaded at: https://www.mdpi.com/article/10.3390/nano13142126/s1, Figure S1: Schematic of the thermal lens setup; Table S1. Thermal-lens measurement parameters; Figure S2: SEM images of graphene oxide with different scales: (a–d) unfractionated, non-purified sample, (e–h) purified and separated fraction with a mass higher than 14 kDa, and (i–l) purified fraction with a mass between 0.5 and 14 kDa. References [97,108,109,110] are cited in the Supplementary Materials.
