# Supplementary material for: The Thermophysical and Physicochemical Properties of the Aqueous Dispersion of Graphene Oxide Dual-Beam Thermal Lens Spectrometry
Source: Nanomaterials (Basel). 2023 Jul 21;13(14):2126. doi: 10.3390/nano13142126 (PMC10385486; doi:10.3390/nano13142126)
Supplement: Supplementary file 1 [file nanomaterials-13-02126-s001.zip › nanomaterials-2480747-supplementary.pdf]

nanomaterials

MDPI

Supplementary Materials

for

Thermophysical and Physicochemical Properties of Aqueous Dispersion of Graphene Oxide Dual-Beam Thermal Lens Spectrometry

Vladislav R. Khabibullin,[1] Daria-Maria V. Ratova,[1] Dmitrii N. Stolbov,[2] Ivan V. Mikheev,[1*] and Mikhail A. Proskurnin [1*]

[1] Analytical Chemistry Division, Chemistry Department, M.V. Lomonosov Moscow State University, d. 1, str. 3, Lenin Hills, Moscow, GSP-1, 119234, Russia; vladhab1995@gmail.com (V.R.K.); darmarrat@gmail.com (D.-M.V.R.); mikheev.ivan@gmail.com (I.V.M.); proskurnin@gmail.com (M.A.P.).

[2] Physical Chemistry Division, Chemistry Department, M.V. Lomonosov Moscow State University, d. 1, str. 3, Lenin Hills, Moscow, GSP-1, 119234, Russia; stolbovdn@my.msu.ru (D.N.S.).

* Correspondence: mikheev.ivan@gmail.com (I.V.M.); proskurnin@gmail.com (M.A.P.); Tel.: +7-495-939-15-68 (I.V.M.), +7-495-939-46-48 (M.A.P.).

Table of Contents

1.	Procedures	2
1.1	Thermal-lens Spectrometer	2
1.2	Shen-Snook model	3
Statistical analysis	3	
2.	Materials morphology characterization.	4
2.1	SEM images	4
References		6

S1. Procedures

S1.1 Thermal-lens Spectrometer

The scheme of the spectrometer is shown in Figure S1.

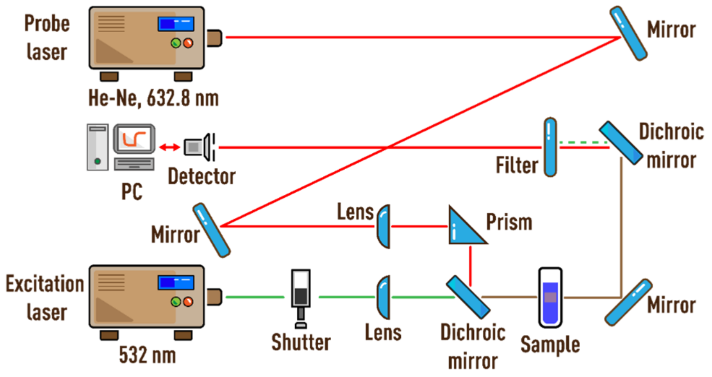

Figure S1. Schematic of the thermal lens setup.

An MGL-FN-532 solid-state laser (wavelength 532 nm, TEM_{00}; Changchun New Industries Optoelectronics Tech. Co., Ltd, Changchun, China) was used as an excitation source. An HNL050L helium–neon laser (wavelength 632.8 nm, TEM_{00}; ThorLabs, Newton, NJ, USA) was used as a probe. The excitation beam passes through a biconvex lens and focuses on the sample in a quartz cell (path length, 10.00 mm), producing a thermal lens. Next, the excitation beam is cut off by a broadband filter. The probe beam passes through the sample and enters the detector (photodiode), where the thermal lens effect is registered as a change in the intensity in the beam center. The excitation and dissipation of the thermal lens are started by an electromechanical shutter (SH05, ThorLabs, Newton, NJ, USA) connected via an analog-to-digital and digital-to-analog converter (c8051Fx–DK, Silicon Labs, Boston, MA, USA) to a personal computer (PC). A single measurement cycle consists of the complete development and dissipation of the thermal lens. Cycle control and signal recording on a PC are carried out using homemade software (C++ programming, Borland Corp., Austin, TX, USA). The power of the laser radiation was controlled using an Optronics Nova II power meter (Ophir Optronics Solutions, Jerusalem, Israel). Measurement parameters are presented in Table S1.

Table S1. Thermal-lens measurement parameters

**Parameter**	**Value**
Excitation laser	
Wavelength, λ_e (nm)	532
Focussing lens focal length, f_e (mm)	200
Confocal distance, Z_{ce} (mm)	10.9
Laser power at the cell, P (mW)	120
Spot size at the waist, ω_{e0} (µm)	42
Probe laser	
Wavelength λ_p (nm)	632.8
Focussing lens focal length f_p (mm)	300
Confocal distance, Z_{cp} (mm)	2.7
Laser power at cell (mW)	4.5
Spot size at the waist, ω_{p0} (µm)	23
Spot size at the cell, ω_p (µm)	520
Other constants	
Cell length (mm)	10
Sample-to-detector distance, Z_2 (cm)	230

Parameter	Value
Mode mismatch factor m	146
Geometric parameters V	36.9
Modulator frequency (Hz)	0.3
Number of transient curves to average	100
Number of experiment repetitions	5

S1.2 Shen–Snook model

To describe the behavior of the probe laser on the detector, the Shen–Snook model for dual-beam thermal lens spectrometry in the stationary state, described in detail in [1], was used. Let us briefly consider the fundamental equations of the model.

The main transient curve equation describing the time-dependent intensity of the probe beam on the detector has the form:

$$I(t) = I(0)\left[1 - \frac{\theta}{2}\tan^{-1}\left(\frac{2mV}{[(1+2m)^2 + V^2](t_c/2t) + 1 + 2m + V^2}\right)\right]^2 \quad \text{(S1)}$$

The probe-beam intensity at each moment $I(t)$ depends on several parameters, including parameters m and V, the thermooptical signal θ, and the characteristic time t_c. $I(0)$ is the intensity of the probe beam at time $t = 0$. The parameter m, also known as the mode mismatch factor, as well as the geometric parameter V of the spectrometer, are described by equations:

$$m = (\omega_{p1}/\omega_{e0})^2, \quad \text{(S2)}$$

and

$$V = z_1/z_c + z_c/z_2[1 + (z_1/z_c)^2], \quad \text{(S3)}$$

where ω_{p1} and ω_{e0} are the radii of the probe and excitation beams in the sample, respectively; z_1 is the distance from the probe beam waist to the sample; z_2 is the distance from the sample position to the detector; and z_c is the probe-laser Rayleigh (confocal) distance.

The thermooptical signal θ is described by the following equation:

$$\theta = \frac{P\alpha l}{k\lambda_e} \times \left(-\frac{dn}{dT}\right), \quad \text{(S4)}$$

where P is the excitation laser power, α is the linear absorptivity, l is the optical path length, k is the thermal diffusivity, λ_e is the excitation laser wavelength, and dn/dT is the temperature coefficient of the refractive index.

To find m, V, and θ, all coefficients and constants, as a rule, are known in advance (from reference data or extra measurements) and the parameters are found using the indicated equations. The characteristic time, on the other hand, is an experimental parameter. The characteristic time is related to thermal diffusivity (D) by the equation:

$$t_c = \omega_{e0}^2/4D, \quad \text{(S5)}$$

Thus, knowing the spectrometer's geometrical and solvent thermal parameters, one can find the target parameter, thermal diffusivity.

Statistical analysis

The relative error is calculated as:

$$\Delta = \frac{X_{mes} - X_{true}}{X_{true}} \times 100\%, \quad \text{(S6)}$$

where X_{mes} is the measured value, X_{true} is the true value (reference or calculated theoretically).

As a measure of reproducibility for thermal diffusivity, the relative standard deviation was used, which is found as follows:

$$S_r = \frac{S}{\overline{D}},\tag{S7}$$

where S is the standard deviation found from the equation:

$$S = \sqrt{\frac{\sum_{i=1}^{n}(D_i - \overline{D})^2}{n-1}},\tag{S8}$$

where D_i is the thermal diffusivity found in a single experiment; $\overline{D}$ is the average thermal diffusivity found as:

$$\overline{D} = \sum_{i=1}^{n} D_i/n,\tag{S9}$$

where n is the total number of measurements. In all experiments, except for the study of the effect of the induction study, the number of measurements was 3–5 times.

S2. Materials morphology characterization

S2.1 SEM images

The SEM images show the layered structure of GO with several overlapping layers, which corresponds to the literature data [2]. In the structure of unseparated GO (Figure S2), small inclusions of tiny highly oxidized fragments (oxidative debris) [3] are visible, due to which there are multilevel cohesive layers in the structure, while samples with mass over 14 kDa and fraction in the range of 0.5 and 14 kDa have separated, thinly folded layers that are closely spaced and do not interact with each other, indicative of rGO morphology [4].

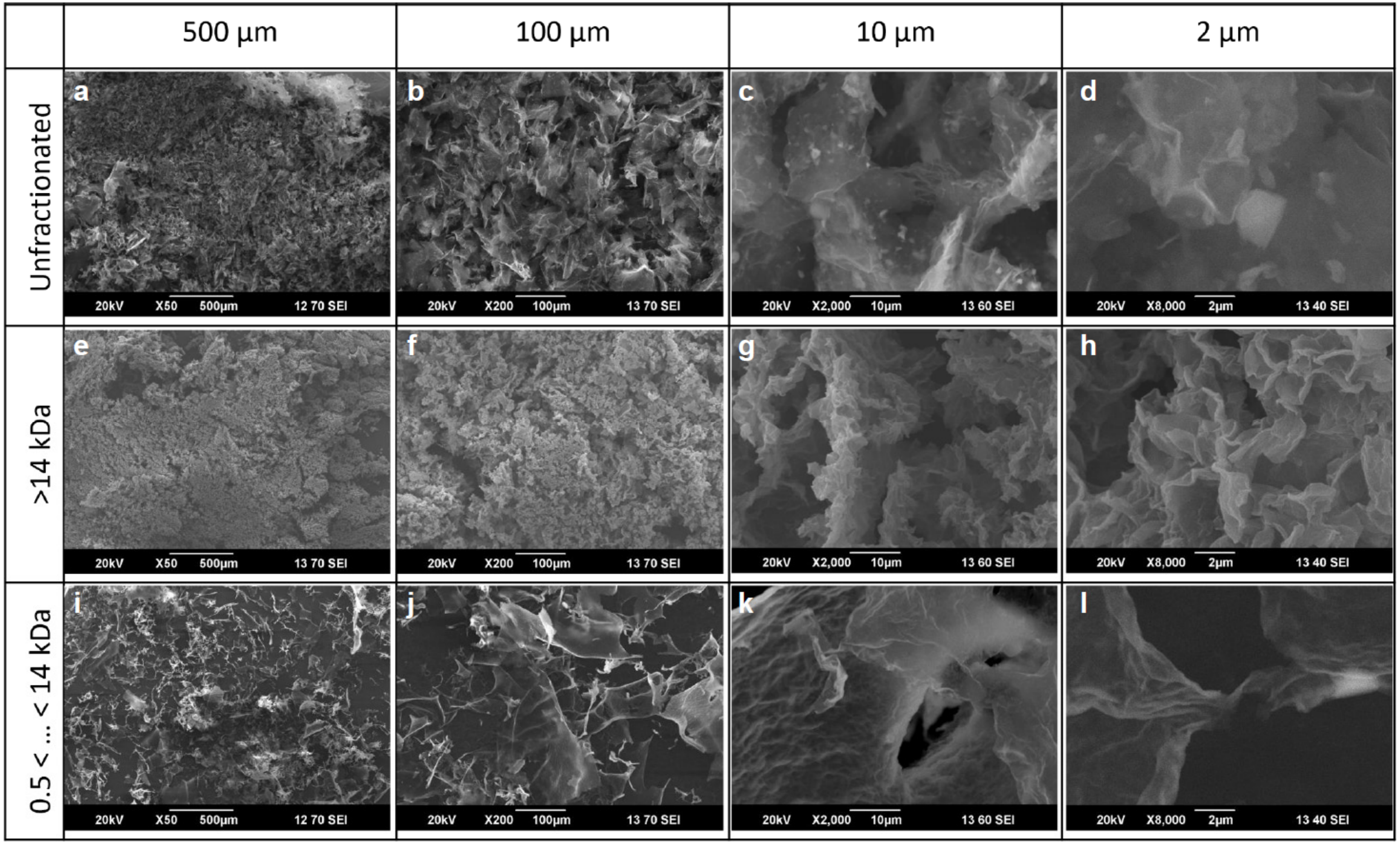

Figure S2. SEM images of graphene oxide with different scales: (a–d) unfractionated, non-purified sample; (e-h) purified and separated fraction with a mass higher than 14 kDa; and (i–l) purified fraction with a mass between 0.5 and 14 kDa.

References

1. Shen, J.; Lowe, R.D.; Snook, R.D. A model for cw laser induced mode-mismatched dual-beam thermal lens spectrometry. *Chem Phys* **1992**, *165*, 385-396, doi:10.1016/0301-0104(92)87053-c.
2. Pattarith, K.; Areerob, Y. Fabrication of Ag nanoparticles adhered on RGO based on both electrodes in dye-sensitized solar cells (DSSCs). *Renewables: Wind, Water, and Solar* **2020**, *7*, 1, doi:10.1186/s40807-020-00058-3.
3. Faria, A.F.; Perreault, F.; Elimelech, M. Elucidating the Role of Oxidative Debris in the Antimicrobial Properties of Graphene Oxide. *ACS Applied Nano Materials* **2018**, *1*, 1164-1174, doi:10.1021/acsanm.7b00332.
4. Valmonte, Z.; Baker, Z.; Loor, J.; Sarkar, A. Concurrent Reduction and Stabilization of Graphene Oxide Dispersion by Silk-Inspired Polymer. *ACS Applied Polymer Materials* **2023**, doi:10.1021/acsapm.3c00353.
